# Surgical flip-dislocation of the bicolumnar approach without olecranon osteotomy versus olecranon osteotomy in type AO 13C3 distal humeral fracture: a matched-cohort study

**DOI:** 10.1186/s13018-023-04405-0

**Published:** 2023-11-29

**Authors:** Shi-Cheng Zhou, Sheng-Yu Jin, Qing-Yu Wang, Guang-Kai Ren, Chuan-Gang Peng, Yan-Bing Wang, Dan-Kai Wu

**Affiliations:** Orthopaedic Medical Center, The Second Hospital of Jilin University, No. 218, Ziqiang Street, Nanguan District, Changchun, Jilin People’s Republic of China

**Keywords:** Surgical flip-dislocation, Olecranon osteotomy, Distal humeral fracture, Type AO 13C3, Propensity score matching

## Abstract

**Background:**

Our experience with the surgical flip-dislocation of the bicolumnar (SFDB) approach for type AO 13C3 humeral fractures indicates that this surgical approach can be performed safely and effectively in appropriately selected patients. We aimed to evaluate the clinical outcomes of the SFDB approach without olecranon osteotomy (OO) for type AO 13C3 distal humeral fractures.

**Methods:**

We retrospectively reviewed 65 cases of type AO 13C3 distal humeral fractures treated between April 2008 and July 2018; 33 patients were treated with the SFDB approach, and the remaining were treated with OO. Propensity score matching was used to control for sex, age, and the American Society of Anesthesiology score. Elbow pain, range of motion, stability, and function were assessed using the Mayo Elbow Performance Index (MEPI) and the Disabilities of the Arm, Shoulder, and Hand questionnaire. Clinical complications, reoperation rates, and radiographic results were compared between the groups.

**Results:**

Operative time and blood loss were significantly lower in the SFDB group than in the OO group (*P *= 0.001, *P *= 0.002, respectively). At the final follow-up, the mean postoperative MEPI did not significantly differ between the groups (*P *= 0.628). According to Morrey's criteria, a typical functional range of elbow motion was achieved in 12 and 15 patients in the SFDB and OO groups, respectively.

**Conclusions:**

The SFDB approach achieves superior exposure of the articular surface without injury to the extensor mechanism in type 13C3 articular surface fracture treatment. This approach also results in good early functional recovery and clinical outcomes, with a low risk of complications.

## Background

Distal humeral fracture is an uncommon injury that affects 5.7 per 100,000 persons yearly [1]. Such fractures are technically demanding in orthopedic trauma surgery, which are comminuted intra-articular distal humerus fractures (type AO 13C3) [2], owing to complex operative complications [3]. Controversy exists regarding the intraarticular distal humerus restoration and fixation approach with minimal disruption to the soft tissue and extensor mechanisms. Anatomical restoration of the articular surface can only be achieved with a good view of the surface. Joint stiffness, pain, and weakness often compromise postoperative joint function. Therefore, minimizing damage to the extensor mechanism is critical for early return to movement and for avoiding joint stiffness.

Different approaches, such as olecranon osteotomy (OO) and triceps-reflecting, triceps-detaching, and triceps-splitting procedures, have been proposed to improve distal humeral fracture treatment. Although OO is one of the most commonly used techniques for achieving good visualization and stabilization of intraarticular fractures, it also causes complications, including healing issues, hardware complications, wound complications, and nerve palsy [4–13].

Schildhauer et al. [14] presented a triceps-saving procedure involving a "two-window" technique for treating distal humeral fractures. This approach preserves the triceps tendon insertion; however, it renders restoration of the articular fracture fragment difficult, especially for the anterior articular surface, compared to the OO approach. New techniques for reducing fracture fragments to restore alignment and the articular surface are crucial to reduce damage to the extensor mechanism and sufficiently expose the articular surface.

In recent years, we have used a modified reduction technique based on the triceps-sparing participial method, the surgical flip-dislocation of the bicolumnar (SFDB) approach, which permits adequate exposure of the articular surface of the distal humerus with less disruption to the extensor mechanism. This retrospective observational study evaluated the clinical and functional outcomes of patients with complex distal humeral fractures (type AO 13C3) treated with SFDB.

The present study aimed to compare the clinical efficacy and value of the SFDB and OO approaches for the surgical treatment of type AO 13C3 comminuted articular surface fractures. We hypothesized that the SFDB approach would provide superior visualization of the distal humerus surface, facilitating the reduction and fixation of fractures.

## Methods

### Participants

**Fig. 1:**
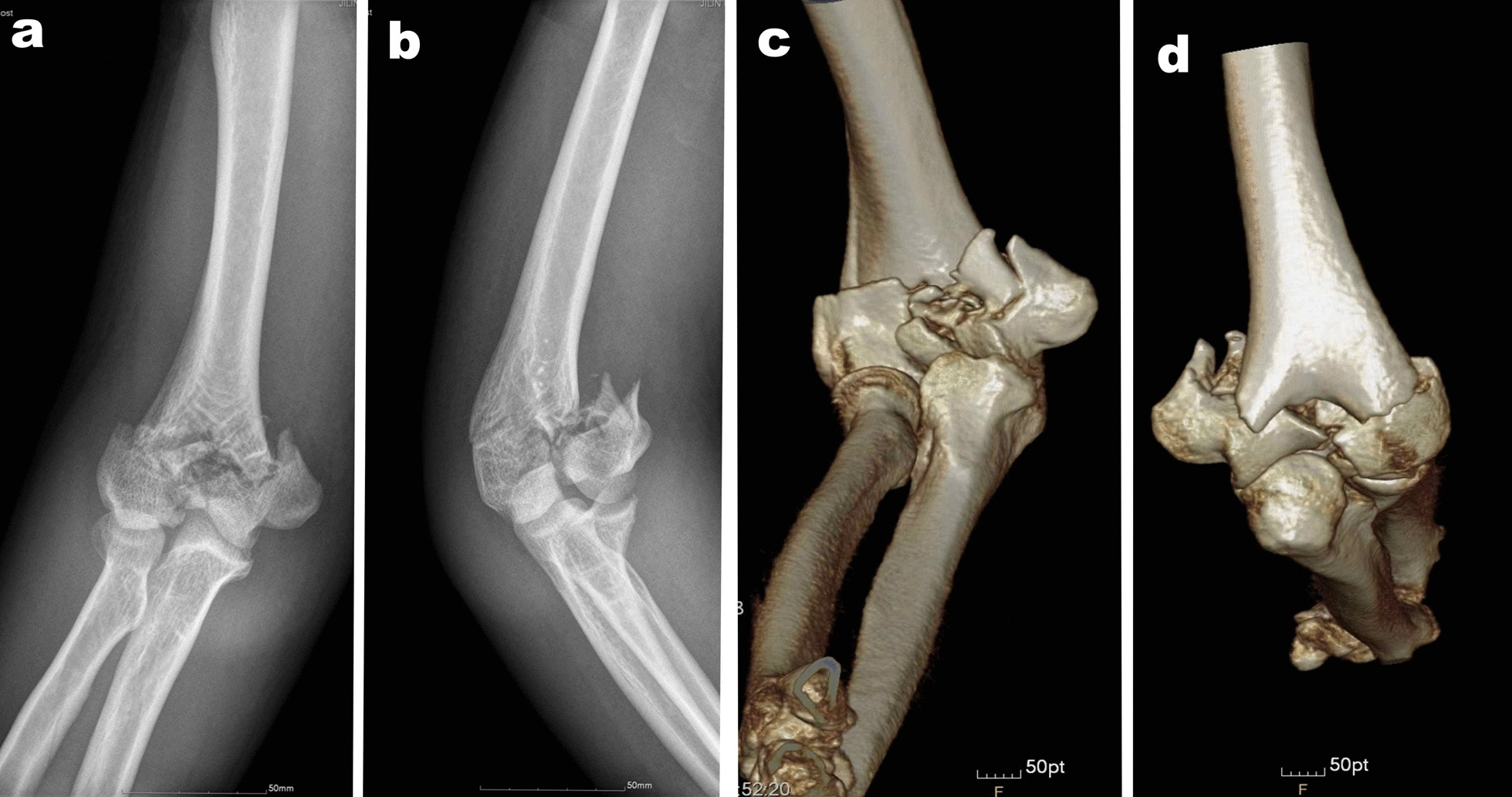
The radiographs of the distal humeral fracture. **a**–**d** X-Rays and reconstructed 3D CT images of AO type13C3 distal humeral fracture

The Ethics Committee of our hospital approved this study, and informed consent was obtained from all participants. This was a single-center, retrospective study of patients treated between April 2008 and July 2018. The diagnosis and classification of distal humeral fractures were verified using radiography and three-dimensional computed tomography (Fig. 1). Sixty-five consecutive patients who underwent the SFDB or OO approach for type AO 13C3 distal humeral fractures were included. Between April 2008 and May 2014, the surgeon in our unit performed OO for all patients with type AO 13C3 distal humeral fractures. In 2014, we found that by dislocating the lateral condyle in patients with simple lateral humeral condylar fractures, it was possible to obtain sufficient visualization of the joint to achieve anatomical reduction of the articular surface fracture. Therefore, we started attempting this approach for type AO 13C3 distal humerus fractures.

The inclusion criteria were age > 18 years, 2018 AO 13C3 intercondylar fracture of the distal humerus using AO/ASIF classification, and achievement of anatomical restoration and internal fixation of type AO 13C3 distal humeral fracture through the triceps-sparing participial approach. The exclusion criteria were severe injury (open fracture), nonunion, or delayed fracture of the distal humerus; pathological fracture of the distal humerus; multiple fractures in the same extremity or a pre-existing joint pathology; and less than 12 months follow-up. One patient was lost to follow-up because she died of cerebral hemorrhage 6 months postoperatively. Thus, 64 patients were finally included. In total, 33 patients underwent surgical flip dislocation using a bicolumnar approach without OO; the remaining patients underwent OO.

### Surgical technique

#### Step 1. Exposure to the fracture

**Fig. 2:**
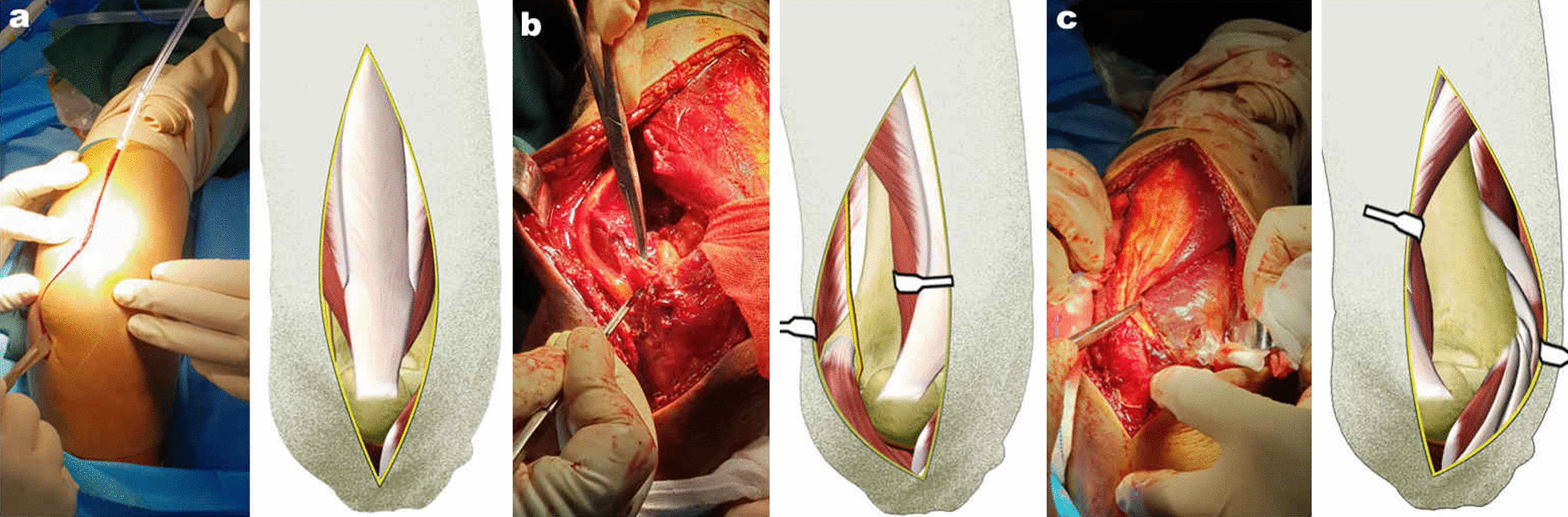
The photograph shows skin incision and dissection. **a** A posterior midline incision of the elbow. **b**–**c** The bilateral borders of the triceps muscle were identified and bluntly split from the intermuscular septum to form medial and lateral windows. The ulnar nerve was identified and protected

One senior orthopedic surgeon performed all procedures. The patient was placed supine, and the shoulder on the affected side was supported by the injured arm resting on the chest. This position allows the elbow to be placed intraoperatively on the receiving end of a C-arm by abducting the affected limb. Fluoroscopic guidance was used for the surgical procedure. The limb was prepared and draped, and a tourniquet was applied to the proximal upper extremity. A posterior midline incision was made around the top of the elbow in the distal portion; if necessary, the incision was extended proximally (Fig. 2).

Full-thickness fasciocutaneous flaps were elevated over the deep fascia. The ulnar nerve was identified and protected. The bilateral borders of the triceps muscle were determined and bluntly split from the intermuscular septum to form medial and lateral windows (Fig. 2). The two windows were connected through the space between the posterior muscles and distal humerus by blunt dissection; muscle stripping was limited to the distal half of the humerus to protect the radial nerve. The triceps muscle was elevated, and the fat pad was excised from the olecranon fossa, which allowed space for straightforward visualization of the posterior articular surface of the distal humerus. However, the anterior articular surface remained obscure, making anatomical restoration of the anterior articular surface challenging.

#### Step 2. Exposure and anatomical restoration of the articular surface of the distal humerus

**Fig. 3:**
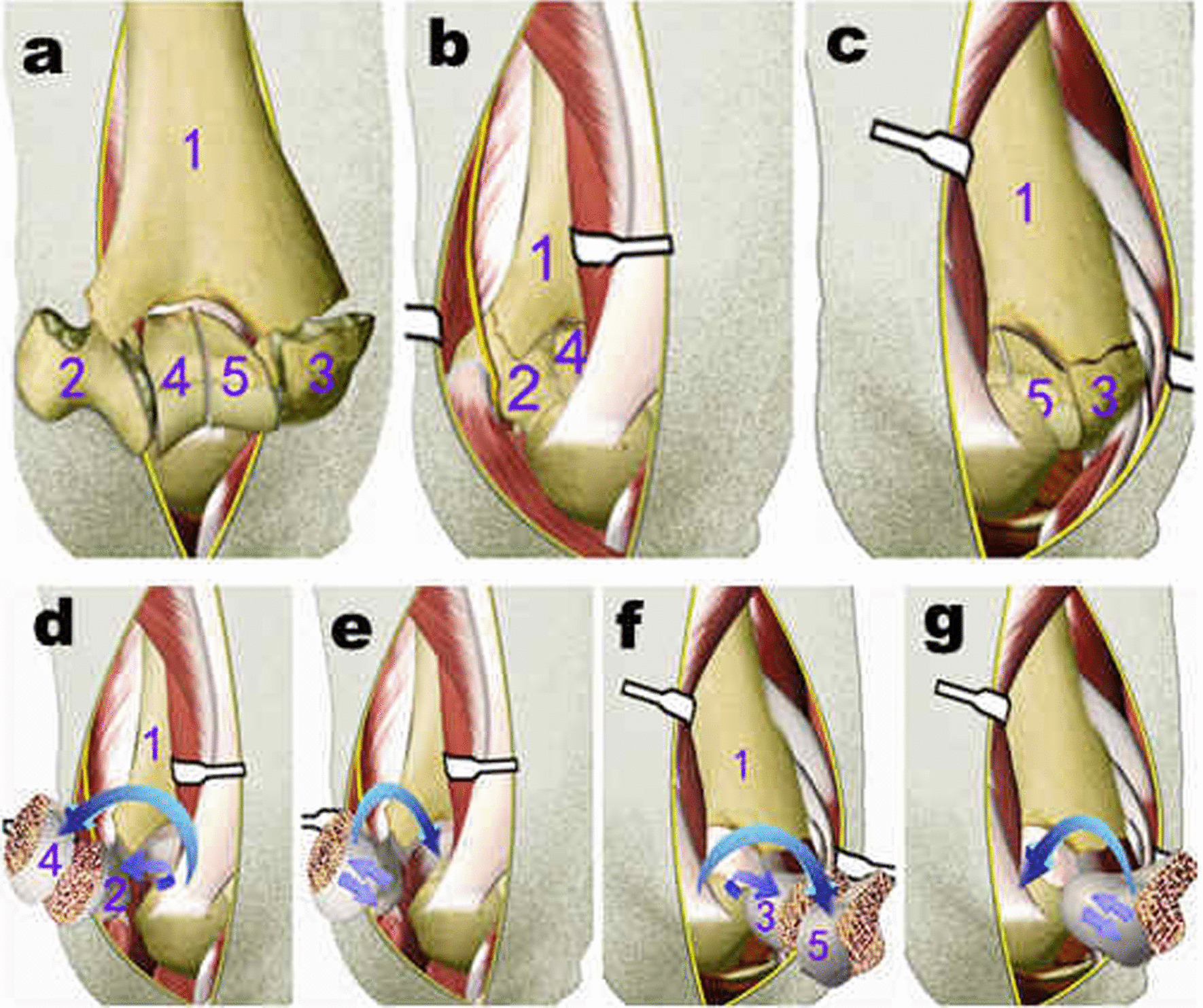
**a** The images of AO type13C3 distal humeru fracture. **b**–**c** The lateral window and the medial window of the fracture site. **d**–**g** First, the medial and lateral condyles fracture fragments were turned 180° through the two-window space, using the collateral ligament as the axis, respectively. Then, the comminuted condyles fracture fragments were anatomically fixed under direct visualization, using a 1.0 mm K-wire for a small fragment or a 2.7 mm screw for a bigger fragment. Finally, the medial and lateral condyles of the humerus were turned back toward the bottom of the triceps muscle and matched with olecranon, and fixed to the humerus shaft

To achieve anatomical restoration of comminuted intercondylar fractures, including anterior articular surface fractures, we used a novel method based on the triceps-sparing participial approach, in which the total articular surface was exposed intraoperatively (Fig. 3).

**Fig. 4:**
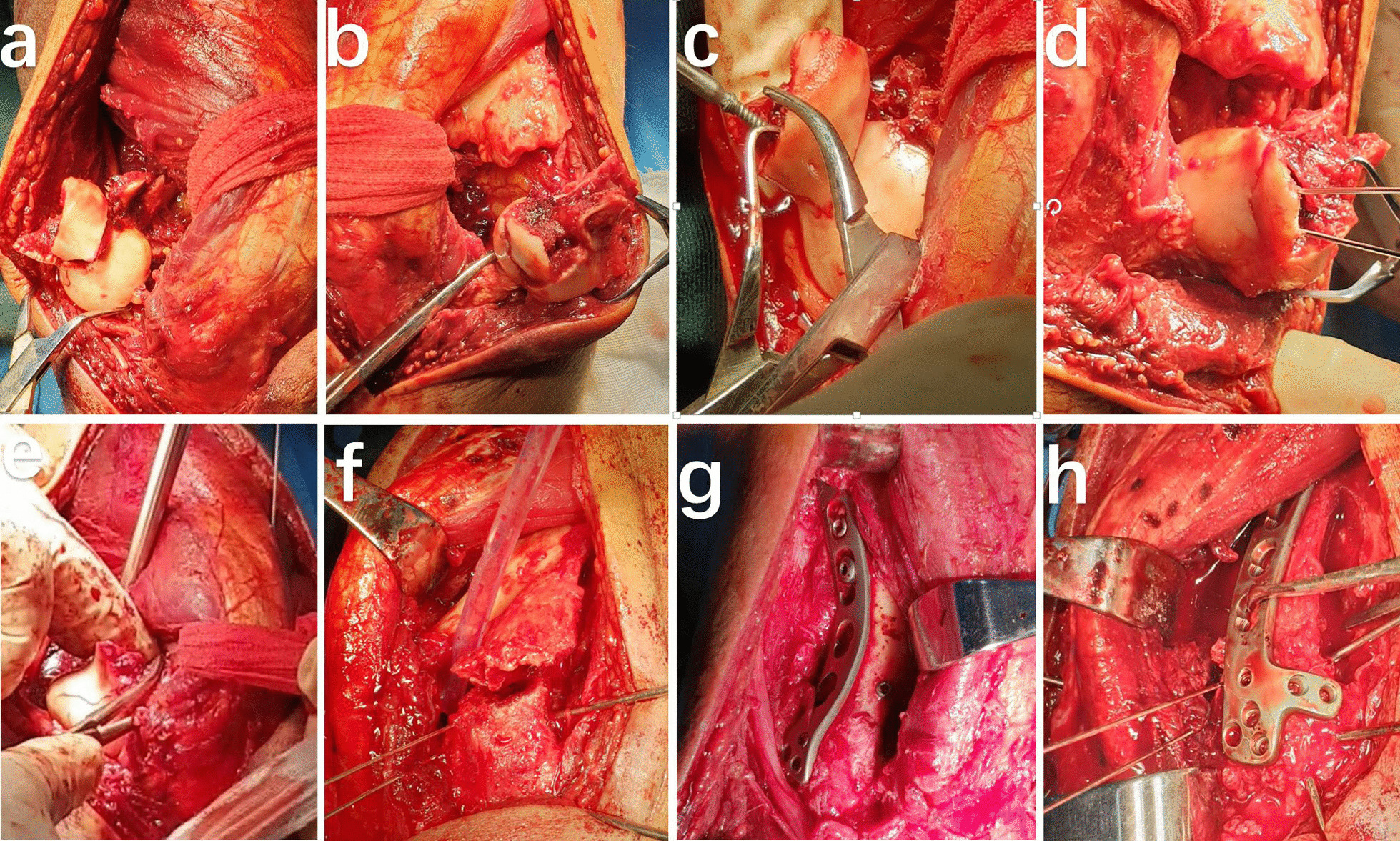
Intraoperative photograph showing the reduction to restore the articular surface without olecranon osteotomy. **a**–**d** exposure of fracture  fragments. **e**–**h** reduction and fixation of fracture fragments

First, the medial and lateral condyles of the humerus were rotated 180° through the two-window space using the collateral ligament as the axis. Following this process, the posterior insertion of the medial collateral ligament was partially cut, if necessary, to obtain a better view of the anterior articular surface of the medial condyle. The medial ulnar collateral ligament (MUCL) complex of the elbow comprises the anterior bundle (AB), posterior bundle (PB), and transverse ligament. The AB is the strongest component of the ligamentous complex and the primary restraint to valgus stress. The PB of the MUCL is a fan-shaped area of capsular thickening that extends from the medial epicondyle to the semilunar notch of the ulna. Following anterior transposition of the ulnar nerve, posterior dislocation of the medial humeral condyle can be easily achieved by extending a longitudinal resection of Osborn's ligament distally. However, in some cases where the soft tissue around the medial condyle is under more tension and it is difficult to dislocate the condyle posteriorly, the PB of the ulnar collateral ligament is resected partially in the insertion of the posterior bundle of MUCL to achieve posterior dislocation of the medial condyle of the distal humerus. The Osborne ligament and posterior bundle are repaired once the fracture has been reduced and fixed. During the partial resection of the medial collateral ligament, we protected the main static stabilizing structure of the AB of the MUCL and the dynamic stabilizing structure of the ulnar carpal flexor due to the anti-rotation stress. Therefore, the stability of the elbow joint was not impaired. Fractures of the humeral trochlea were simultaneously removed and cleaned. This method exposes the anterior articular surface, with complete exposure of the articular surfaces of the humeroulnar and humeroradial joints (Fig. 4).

Additionally, the comminuted fracture fragments were anatomically reduced to either side of the condyle and fixed through direct visualization using a 1.0-mm K-wire for small fragments or a 2.7-mm screw for large fragments. A complicated type AO 13C3 intercondylar fracture was then simplified to a type C2 simple intercondylar fracture. Finally, the medial and lateral condyles of the humerus were turned back to fix the distal humeral fragment.

For some complex and challenging cases, the reduction of medial column fragments was performed first, and the medial column was then temporarily fixed on the olecranon ulna with a 1.5-mm Kirschner wire, using the humeroulnar joint as a reference. The humeroulnar joint, radial head, and absent lateral column formed a ring through which the reduction and fixation procedures could be assisted. Following anatomical restoration, the medial and lateral condyles were temporarily fixed with 2.0-mm Kirschner wires under fluoroscopic guidance. Using this technique, a complex C3 fracture was transformed into a C2 fracture.

#### Step 3. Fracture reduction and internal fixation

The medial and lateral columns of the distal humerus were reconstructed using the orthogonal plating technique, with one plate on the dorsolateral side of the humerus and the other on the medial column (Fig. 4). Direct visualization and fluorescence were used to confirm the reduction, alignment, and length of the screws. The stability of fracture fixation and elbow range of motion (ROM) was assessed intraoperatively. A surgical drain tube was placed beneath the triceps muscle and wound during surgery. The fascia profunda, subcutaneous fat, and skin were sutured layer by layer. Anterior transposition of the ulnar nerve was performed in all patients.

### Postoperative protocol

To achieve early functional restoration, passive and active movement of the elbow joint was performed as soon as possible. Per the patient's general condition, dynamic exercises of the shoulder, wrist, and fingers were started on the day of surgery, and functional movements of the elbow joint were started on the day after surgery. Indomethacin was administered orally (25 mg, three times per day) for 6 weeks from the day after surgery. After removing the drainage tube, fully active and assisted elbow motions were initiated. Implant removal could be performed 1–2 years after the remodeling. Routine metal removal was not recommended because although younger patients may require implant removal, older patients may tolerate it.

### Assessments

**Fig. 5:**
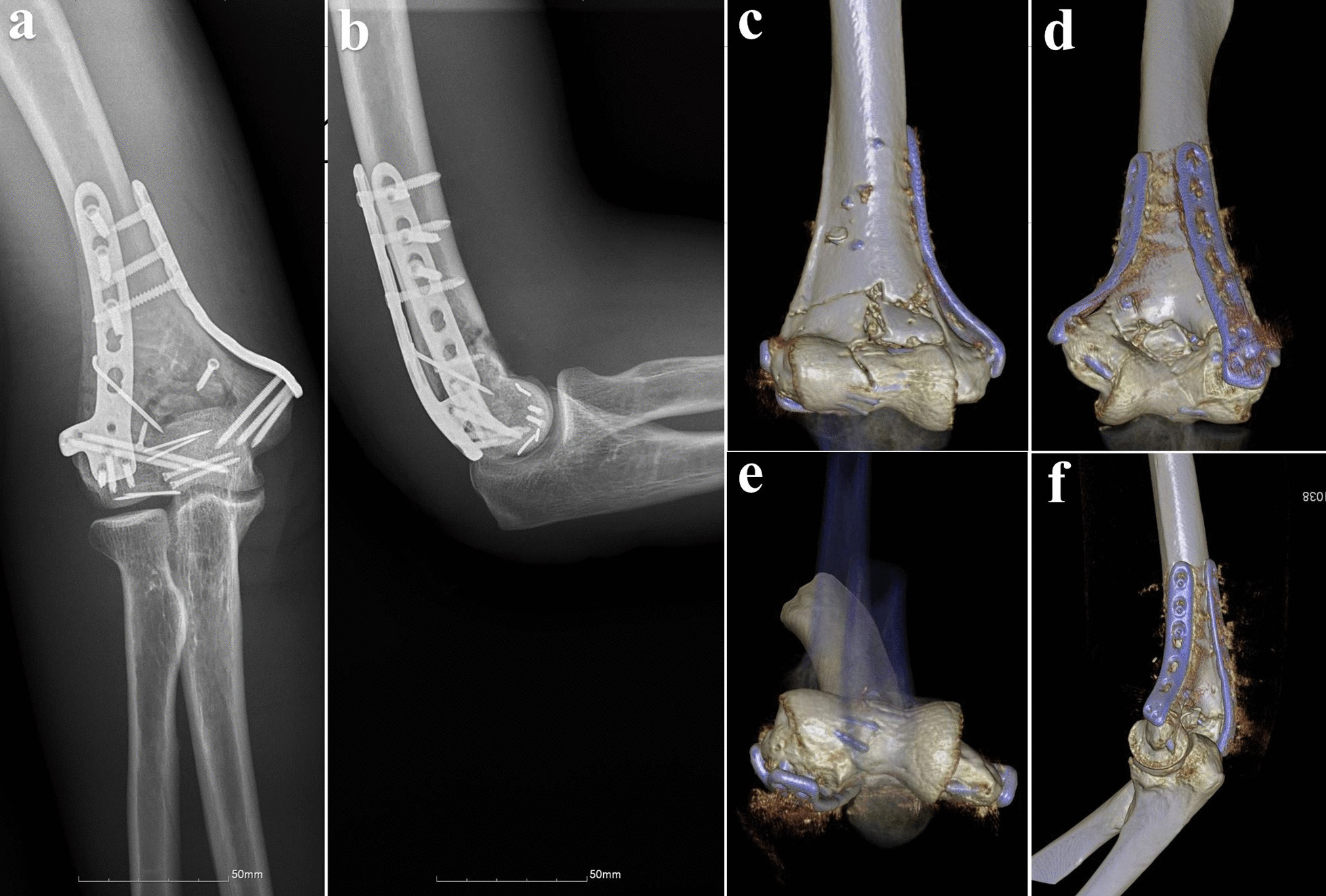
The postoperative radiographs were shown during the follow-up. **a**–**d** Postoperative X-Rays and postoperative reconstructed 3D CT images of AO type13C3 distal humerus fracture

**Fig. 6:**
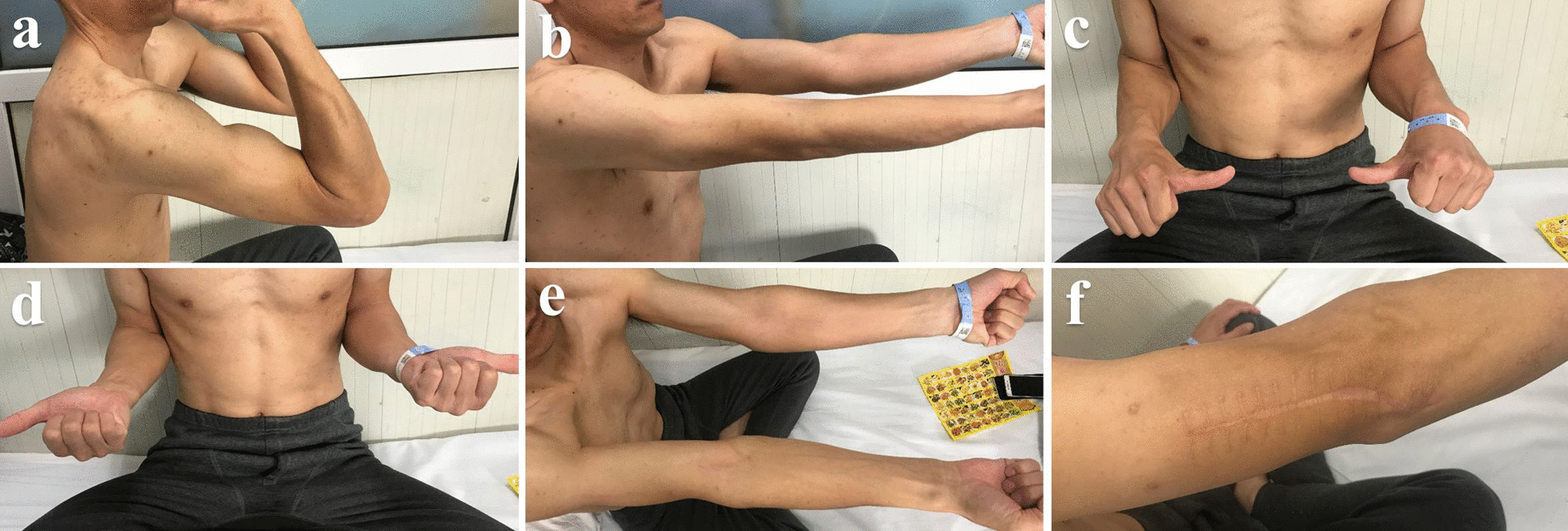
No significant limitation in flexion, extension, pronation, and supination of the left elbow at 1-year follow-up. **a** Flexion, **b** extension, **c** pronation **d** supination function of the injured limb. **e**–**f** the showing of the wound incision

All patients underwent routine evaluations, including clinical and radiographic assessments and ROM evaluations, at 1, 3, 6, and 12 months postoperatively and once yearly thereafter (Figs. 5 and 6).

The fracture healing time and incidence of complications, including delayed union, nonunion, malunion, and heterotopic ossification, were recorded. The range of daily elbow motion was evaluated using Morrey's criteria [15], which includes 30° extension to 130° flexion, > 50° pronation, and > 50° supination at the final follow-up. Elbow pain, ROM, stability, and function were comprehensively assessed using the Mayo Elbow Performance Index (MEPI). The maximum MEPI score totals 100 points, with a 90–100 score indicating an excellent result; 75–89, a good result; 60–74, a fair result; and < 60, a poor result [16]. Triceps muscle strength was assessed subjectively, with comparison to the uninjured arm, and muscle strength was graded as usual, good, or fair [17, 18]. Disabilities of the Arm, Shoulder, and Hand (DASH) questionnaire scores were obtained during follow-up [19].

### Statistical analysis

Propensity score matching was used to minimize bias due to possible confounding factors. The SFDB and OO groups were propensity score-matched in a 1:1 ratio, based on age, sex, body mass index, and American Society of Anesthesiology (ASA) score, using the propensity scores generated by logistic regression. After matching, all clinical and functional outcomes were compared between the groups using the paired t test. Before starting this trial, we performed a power analysis to estimate the sample size. The sample size in this study was sufficient to detect a significant difference between the cohorts at an alpha value of 0.05 and a power of 80%. Statistical analysis was performed using IBM SPSS Statistics (version 22, New York, NY, USA). Statistical significance was set at *P *< 0.05.

## Results

### Clinical and radiological results

Demographic data are shown in Table 1. The average age was 45.8 (22–76) years. There were 28 men (28 elbows; 43.8%) and 36 women (36 elbows; 56.2%). Causes of the high-energy injuries included traffic accidents and falls. All patients were followed up for an average of 16.9 months (Table 1). The mean operative duration was 112.0 and 136.5 min for the SFDB and OO groups, respectively (*P *< 0.001). Implant removal was performed in 20 (74.1%) and 25 patients (74.1%) in the SFDB and OO groups, respectively. On postoperative radiological assessment, all cases were reset with no more than a 2-mm step-off and 5° malalignment. Bone union was achieved in all cases, and the mean time before radiological union was 3.4 and 3.3 months in the SFDB and OO groups, respectively (*P *> 0.05).

**Table 1:** Demographic data of the two matched groups

	SFDB group	OO group	*P*
Age (range, years)	45.2 (23–76)	46.3 (23–76)	
Sex (male/female)	15/12	15/12	
Body mass index	24.2 ± 2.8	24.0 ± 2.2	0.834
American Society of Anesthesiology	2 (1–3)	2 (1–3)	0.06
*Fracture type (range, AO)*			
13C3	27	27	
*Injury side*			
Right	16	14	-
Left	11	13	-
*Injury mechanism*			
Fall on grand	10	8	-
Fall from height	12	11	-
Vehicle collision	5	8	-

### Functional results

At the final follow-up, the mean MEPI score was 86.4 ± 9.8 and 85.2 ± 13.2 in the SFDB and OO groups, respectively (*P *> 0.05) (Table 2).

**Table 2:** Details of clinical and functional outcomes of the two matched groups

	SFDB group	OO group	*P*
Follow-up duration (range, months)	16. 9 (14–26)	17.0 (15–26)	0.900
Operation time (range, mins)	112.0 (109–137)	136.5 (126–157)	< .001
Perioperative blood loss (mL)	193.7 ± 88.6	276.7 ± 101.2	0.002
*Range of motion at final follow-up*			
Flexion (°)	118.3 ± 12.4	116.7 ± 10.4	0.616
Extension (°)	11.1 ± 3.5	13.1 ± 5.2	0.174
MEPI	86.4 ± 9.8	85.2 ± 13.2	0.628
DASH score	27.7 ± 6.7	29.6 ± 6.2	0.285

According to Morrey's criteria, a normal functional range of elbow motion was achieved in 12 and 15 patients in the SFDB and OO groups, respectively. Additionally, the mean postoperative DASH score was 27.7 ± 6.7 and 29.6 ± 6.2 in the SFDB and OO groups, respectively (*P *= 0.285) (Table 2).

### Complications

No case of infection, elbow instability, dislocation, delayed union, nonunion, or malunion was observed in the SFDB group. No implant failures or prominences were observed. Anterior or posterior heterotopic ossification was observed on imaging in two patients (7.4%) (Table 3). One patient (3.7%) experienced ulnar neuropathy postoperatively, and the patient recovered gradually over subsequent follow-up visits.

**Table 3:** Comparison with previously published studies

Author	No	Fracture AO/ASIF classification	Range of motion(°)	MEPI	DASH	Nerve palsy	HO	Infection	Delayed union, nonunion, malunion	Implant prominence	Stiffness
Gang Chen et al*.* [11]	33	C1,C2,C3	121.9 ± 19.4/10.9 ± 13.2	84.5 ± 15.5	–	2	4	1	2	–	–
Jwalant Patel et al*.* [12]	31	C1,C2,C3	115.8 (range 85–150)/19 (range 5–35)	87.9	–	1	–	–	–	3	–
Weber et al*. *[20]	15	C1,C2,C3	116.1 ± 14.7/23.2 ± 12.70	–	23.2	5	5	0	5	2	12
Ansari MF et al*.* [21]	28	C2	106.8 ± 10.1/12.9 ± 8.3	83.57 ± 10.96	35.32 ± 8.89	5	3	3	2	3	–
İbrahim Azboy et al*.* [22]	44	C1,C2,C3	–	83.5 (range 55–100)	20.1 (range 4–57)	2	–	14	3	4	–
Rahul Singh MS et al*. *[23]	24	C1,C2,C3	123.75 ± 9.69/9.37 ± 7.70	87.08 ± 11.87	–	2	1	2	0	5	3
Byung-Sung Kim et al*.* [24]	55	C2,C3	130.0 ± 9.0/ − 0.7° ± 2.6°	85.7 (range,70–100),	20.0 (range, 0–68)	6	6	–	3	–	10
Present study	27	C3	118.3 ± 12.4/11.1 ± 3.5	86.4 ± 9.8	27.7 ± 6.7	1	2	None	None	None	–

*MEPI* Mayo Elbow Performance Index, *DASH* The Disabilities of the Arm, Shoulder and Hand questionnaire, *HO* Heterotopic Ossification

No case of delayed union, nonunion, implant failure, or implant prominence was noted in the OO group. One patient had limited flexion but refused additional surgery because he considered his functional state acceptable. Anterior or posterior heterotopic ossification was observed on imaging in five patients (18.5%); four had limited ROM, and the remaining patient had poor function in multiple directions and is awaiting ligament release during hardware removal surgery.

## Discussion

The present study found excellent outcome scores with the SFBD approach for type AO 13C3 distal humeral fractures, with a low revision rate for severe conditions. The SFBD approach is a reliable treatment option for complex distal humeral fractures based on the present data.

This study proposed a modified reduction approach based on the triceps-sparing participial method. In this method, the medial and lateral condyles of the humerus were turned outward 180° through medial and lateral windows to expose the hidden anterior articular surface. This method exposes the anterior articular surface, and the articular surfaces of the humeroulnar and humeroradial joints can be exposed without disrupting the extensor mechanism. This technique is better than common operative methods, such as OO, triceps-reflecting, and triceps-splitting approaches. A cadaveric study by Wilkinson et al. [20] demonstrated that the fraction of exposed articular surface for triceps-splitting, triceps-reflecting, and OO approaches was 35%, 46%, and 57%, respectively.

OO is the most commonly used method for treating distal humeral fractures because it provides wide exposure to joint surfaces. However, several osteotomy-related complications can occur with reconstructing the olecranon, such as osteotomy healing issues (delayed union and nonunion), hardware failure (prominence of the screws or plates), irritation, infection, elbow joint stiffness, and heterotopic ossification, which may require additional surgery (Table 3) [12, 13, 21–25]. Similarly, the triceps-splitting approach does not adequately expose the articular surface compared to other procedures, as demonstrated in the above-mentioned cadaveric study [20], and this approach is accompanied by complications, such as fibrosis, intermuscular nerve branch injury, and muscle weakness caused by direct muscle trauma. However, these restoration-related difficulties are overcome by the SFDB approach.

The SFBD approach takes full advantage of the type AO 13C3 intercondylar fracture line and provides adequate exposure of the distal humeral articular surface without the requirement of additional osteotomies. The SFBD approach also reduces further damage to the soft tissues and blood supply around the fracture end, allowing early healing of the fracture. This approach utilizes a relatively bloodless plane and avoids direct injury to the extensor mechanism. It also reduces scar formation due to the extension of the incision, preventing functional disturbances due to the disruption of the triceps muscle insertion. In addition, we observed a significantly lower risk of postoperative complications than those in other studies (Table 3). In the present study, one patient (3.7%) in the OO group experienced ulnar neuropathy postoperatively, and the patient recovered gradually. There was also a case of poor multidirectional function due to heterotopic ossification due to reoperation and metal removal surgery. Based on our observations, the SFDB group had better functional and clinical outcomes and lower complication rates than the OO group.

Through years of experience, we have found that not every fracture requires pre-dislocation of both columns; for simple intercondylar fractures of the distal humerus, reduction can often be accomplished by selecting either side of the column for pre-dislocation. In complex cases of distal humeral fractures, a relatively intact medial column can be obtained by first fixing and reducing most of the fracture fragments to the medial condyle. Pre-dislocation of the fracture line splicing between the medial and lateral columns as close to the lateral side as possible facilitates the conversion of C3 fractures to C2 fractures. Using the SFBD method and the ulnar olecranon joint surface as a reference, we obtained anatomical reduction of the posterior and distal articular surfaces of the distal humerus.

The present study has some limitations. First, this study was designed as a retrospective case series, and the number of patients was small. Furthermore, the study is limited by the short follow-up period, lack of objective muscle strength testing, and inability of some patients to adhere to the rehabilitation guidelines.

## Conclusion

Compared with the OO approach, the SFDB approach is highly efficient for reducing the articular surface in type C3 distal humeral fractures, with a low complication rate and a stable procedure for surgical intervention. However, large randomized controlled trials comparing different approaches and fixation methods are required to identify the optimal treatment protocol for type C3 distal humeral fractures.

## Data Availability

The data sets supporting the results of this article are included within the article and its additional files. The datasets are available from the corresponding author on reasonable request.

## Abbreviations

SFDB: Surgical flip-dislocation of the bicolumnar
OO: Olecranon osteotomy
MEPI: Mayo Elbow Performance Index
ROM: Range of motion
DASH: Disabilities of the Arm, Shoulder, and Hand
ASA: American Society of Anesthesiology Score
VAS: Visual analog score
HO: Heterotopic ossification
